# Who defines the question in neglected tropical disease research?

**DOI:** 10.1371/journal.pntd.0014836

**Published:** 2026-10-30

**Authors:** Augusto Magubeia Francisco, Mariana Gomes Lima, Sónia Maria Enosse, Clélia Christinha Mello Silva, Ronaldo de Carvalho Augusto

**Affiliations:** 1 Instituto Nacional de Saúde (INS/MISAU), Maputo, Mozambique; 2 National Reference Service for Schistosomiasis, Laboratory of Helminthology and Medical Malacology, Fiocruz Minas, Belo Horizonte, Brazil; 3 LAPSA, Instituto Oswaldo Cruz (FIOCRUZ/IOC), Rio de Janeiro, Brazil; 4 IHPE, Univ Perpignan Via Domitia, CNRS, IFREMER, Univ Montpellier, Perpignan, France; George Washington University Medical Center, UNITED STATES OF AMERICA

## Abstract

Neglected tropical diseases (NTDs) are often framed through pathogens, interventions, and elimination targets, but the systems that produce knowledge about them receive less scrutiny. This Viewpoint argues that unequal control of funding, authorship, and publication shapes NTD research questions and can weaken their public-health relevance. Co-design should begin before protocols are fixed and should be supported by proximity to endemic settings, their institutions, and their lived realities. We propose that education, reciprocal mobility, co-supervised training, and durable partnerships among low- and middle-income countries (LMICs) and high-income countries (HICs) be recognised as research infrastructure, and that funders enable researchers in endemic countries to lead such partnerships directly. NTD research should be judged not only by scientific outputs, but also by who defined the question and what relationships made that question possible.

## Who sets the question?

Neglected tropical diseases (NTDs) are usually described through pathogens, clinical consequences, and epidemiological maps. Less often discussed is the system that produces knowledge about them. Disease burden is concentrated in endemic countries, but authority to define priorities, distribute resources, validate methods, and turn fieldwork into academic capital often remains elsewhere. This pattern has deep roots. Galtung described as scientific colonialism a process in which the centre of gravity for producing knowledge about a place lies outside that place, with data exported for processing elsewhere much as raw materials once were [1]. Formal colonial rule has ended, but this division of labour persists whenever endemic settings supply samples and data while external institutions retain interpretive authority. This asymmetry is not only an ethical concern. It shapes the questions that are asked, the questions that remain invisible, and the likelihood that research will improve the lives of people affected by NTDs.

The WHO road map for NTDs places country ownership at the centre of control, elimination, and eradication (Box 1). Research ownership is less secure [2]. By ownership we mean a fair share of authority over what is studied, how, and why, not exclusive control. Many projects begin far from the places where transmission occurs: calls are written in high-income institutions, methods are selected according to technologies available in well-resourced laboratories, and field sites are identified after the central hypothesis has been fixed. Local teams may then be asked to make a project feasible without participating in the decision about whether it asks the right question. In NTD research, relevance is part of rigour. A molecular, genomic, or diagnostic study can be technically strong yet have limited public-health value if it does not reflect local transmission ecology, health-service constraints, or the policy decisions that its evidence is expected to inform.

**Box 1. Glossary**

Country ownership: the principle, central to the WHO NTD road map, that endemic countries lead their national NTD programmes, set their priorities, and increasingly finance them.

Research ownership: a fair distribution of authority over a research agenda, including who defines the question, controls the budget, interprets the results, and holds leading authorship positions. It does not imply exclusive control by any one party.

Epistemic authority: the recognised standing to produce, validate, and interpret knowledge; in practice, whose claims count as evidence.

Epistemic injustice: a wrong done to someone in their capacity as a knower, for example when their testimony is given less credibility because of who they are or where they work.

Scientific colonialism: a division of scientific labour in which knowledge about a place is produced and controlled from outside it, while local settings supply data and samples.

Mirror journal: an open-access twin of an established subscription journal that shares its editorial board and standards but is financed through article processing charges.

Triangular cooperation: a partnership in which two or more LMICs pursue shared goals with support from a high-income country or a multilateral organisation.

## How authority is produced

Financial asymmetry is a central mechanism through which epistemic authority is produced. Funding for neglected disease research comes largely from a small number of sources in high-income countries (HICs): in 2023, the largest public funder and the largest philanthropic funder together provided more than 60% of the US$4.2 billion invested globally in neglected disease basic research and product development [3]. This support has made much NTD science possible, but it also means that funders’ priorities shape research priorities, and that research effort remains concentrated in high-income regions and only loosely aligned with where disease burden falls [4]. In partnerships between HICs and low- and middle-income countries (LMICs), institutions in endemic countries often enter collaborations after objectives, budgets, and methods have largely been settled [5]. Similar patterns appear outside health: in HIC–LMIC partnerships on energy, technology, and enterprise funded by the UK Global Challenges Research Fund, the language of theory of change and responsible innovation did not guarantee that projects met end-user needs [6]. This is less a matter of intent than of structure: whoever holds the budget tends to shape the question. Changing this dependence requires funders, and the researchers they support, to treat the development of local scientific authority as an explicit objective rather than a by-product of collaboration.

Authorship makes this hierarchy visible. Research conducted in Africa has repeatedly shown limited leadership by investigators from the countries where the work takes place and, in some cases, no local co-authorship [7]. First and last authorship are not merely symbolic positions; they influence promotion, grant eligibility, and institutional prestige [8]. Yet ethics navigation, field logistics, community engagement, language mediation, sample collection, and interpretation of local context are frequently treated as operational support rather than intellectual contribution. This arrangement is inequitable, but it also weakens knowledge production. NTD transmission is shaped not only by pathogen biology but also by social relations, environmental change, health systems, and cultural practices.

Publication systems add another filter. Open access is essential for NTD research, but article processing charges can make openness conditional on wealth. Analyses of Elsevier’s mirror-journal system found lower geographic diversity among open-access authors and no mirror-journal articles led by authors from low-income countries [9]. The resulting cycle is then mistaken for merit: researchers with larger grants publish more visibly, visibility strengthens credibility, and credibility attracts further resources. Such cumulative advantage operates throughout science, but here it compounds with geographic and institutional boundaries, so that the starting point is set less by individual merit than by where a researcher works.

## Co-design requires proximity

These dynamics are the material basis of epistemic injustice: they affect who is, and who is not, recognised as a knower, whose testimony is credible, and which interpretive frameworks count as rigorous evidence [10,11]. A local researcher’s account of why a treatment campaign failed, why a water-contact site matters, or why a diagnostic strategy is impractical can be discounted as anecdotal, while a model developed far from the field is treated as generalisable knowledge. The consequences extend beyond recognition. When local actors do not help define research agendas, important dimensions of transmission, vulnerability, care-seeking, territorial dynamics, and health-system constraints may never become data. What is not measured rarely enters policy debates.

Co-design is often offered as the remedy, but the term has become too elastic. Consultation, employing local staff, or adding a local principal investigator after funding has been secured is not co-design. Genuine co-design begins before the research question is stabilised. It requires affected communities, public-health authorities, and researchers embedded in endemic settings to participate in defining the problem, not only in implementing a protocol. Recent work has argued that meaningful co-design depends on proximity: sustained engagement with the contexts in which marginalisation, resilience, and health needs are lived [12,13]. Long-term community engagement shows what proximity can build. In a citizen-science project on snail-borne schistosomiasis in Uganda and the Democratic Republic of Congo, residents trained as local snail experts and communication ambassadors sustained participation for years and gained recognition in their communities [14]. Such locally trained leaders can carry a project’s knowledge beyond its funding cycle and become reference points for future work on related problems. The concept of used territory makes this concrete: territory is not a neutral surface but space as it is appropriated, worked, and inhabited by the people who use it [15]. Endemic settings are, in this sense, lived territories shaped by culture, labour, infrastructure, water use, mobility, ecological change, and institutional history. A research question that ignores this territorial reality can be technically rigorous and still miss the conditions that sustain transmission.

The same logic applies to basic science. Laboratory work on parasites, vectors, host immunity, drug response, and transmission biology can be foundational for future control. But laboratory science is also shaped by choices: which parasite population is studied, which vector phenotype is prioritised, which exposure is modelled, and which endpoint is interpreted as meaningful. A parasite genome, snail colony, or mosquito strain is not only a laboratory object; it is part of a transmission system embedded in infrastructure, ecology, livelihoods, and policy. Basic research need not promise immediate application, but a laboratory question should be able to state which needs of affected populations it may ultimately serve. The persistent mismatch between research effort and disease burden [4] suggests that this question is too rarely asked, and that research teams’ interests can outweigh the needs of the people studied.

## Education is research infrastructure

Trust is another undervalued infrastructure. Research in endemic settings depends on relationships built over years among communities, health workers, local authorities, and scientific institutions; these relationships enable participation, sample collection, surveillance, and intervention uptake. Trust is not an unlimited resource: partnerships that extract data without strengthening local capacity erode the relationships on which future research and public-health action depend.

Education should therefore be treated as research infrastructure, not as a charitable appendix to externally designed projects. Real co-construction cannot be produced at the moment of proposal writing alone. It requires durable networks through which students, researchers, public-health officers, laboratory teams, and community actors circulate across institutions before grant questions are fixed. Reciprocal mobility, co-supervised degrees, joint field schools, shared data-analysis workshops, mentorship, and small seed grants create time to understand transmission ecology, health-service constraints, institutional histories, and the actors who must turn knowledge into action. In this sequence, the project emerges after the relationship, not before it.

Education programmes linking LMICs, alone or with an HIC partner (triangular cooperation), provide one practical pathway. Postgraduate programmes developed through needs assessment in Mozambique show how training can be linked to institutional strengthening in resource-constrained settings [16]. Brazil-Mozambique cooperation through Fiocruz-linked programmes offers a related example of postgraduate education designed around public-sector needs [17]. Such initiatives should not be considered peripheral capacity building. Nor should they become a parallel circuit. Networks confined to LMICs risk reproducing exclusion in reverse and isolating researchers in endemic countries from expertise, infrastructure, and funding concentrated elsewhere. Their value lies in strengthening the position from which those researchers negotiate with partners in HICs, not in replacing that negotiation.

Researchers in endemic countries can also initiate collaborations with HIC partners rather than wait to be invited into them, co-developing proposals, study design, implementation, and publication under explicit agreements on data, authorship, and intellectual property. The Sub-Saharan African Network for TB/HIV Research Excellence (SANTHE), an African-led consortium funded through the DELTAS Africa initiative with support from Wellcome and the UK government, trained 105 researchers across five countries within five years [18]. Moreover, the Tackling Infections to Benefit Africa (TIBA) partnership, funded by the UK National Institute for Health and Care Research, shows that this is possible in NTD research: its agenda is set in Africa, and 77% of its publications have Africa-based first and/or last authors [19].

## A practical agenda

Several changes follow. Funders should support long-term educational infrastructure for NTD research, including return grants and seed funding that allow trained researchers to develop projects from their own institutions. Funding schemes should enable institutions in endemic countries to lead grants directly, with budget authority, indirect costs, and infrastructure investment. Experience from long-term partnerships in Ethiopia, Uganda, Lao PDR, and Vietnam shows that funders can shift power by directing leadership roles and primary funding to the institutions where research takes place [20]. This is achievable: funders in the United Kingdom, France, Sweden, and Brazil, among others, already combine strategic calls with endemic countries and support for structures designed to last, from Africa-led research partnerships and joint international laboratories to university partnerships sustained over decades [19,21–23]. Journals should require brief reflexivity statements describing how the research question was defined, how local leadership was recognised, and how authorship positions were assigned. Research assessment should reward the field-building work on which NTD science depends, including surveillance capacity, diagnostic evaluation, clinical-trial platforms, biobanks, community trust, training, and policy uptake, not only papers and citations.

None of this requires rejecting international collaboration. NTD research needs technical expertise from institutions outside endemic regions, and the aim is shared authority, not a transfer of control from one region to another. A bidirectional decolonial perspective supports this position: it rejects a simple geographic reversal of authority and asks how colonial and racialised structures shape knowledge production in all settings [24]. Research ownership, as used here, means agency rather than exclusion: researchers and institutions closest to the burden of disease should have substantive power to co-define questions, resources, methods, interpretation, and benefits. The issue is not whether external researchers should participate, but on what terms. External expertise should help answer questions defined together with the people and institutions closest to the burden of disease; it should not arrive with the question already settled. The next phase of NTD research should therefore be judged by two tests: who had the power to define the question before data collection began, and what educational and institutional relationships made that question possible? Co-design becomes meaningful when it is supported by the long-term relationships needed to produce better science.
